# Orienteering Is More than Just Running! Acute Effect of Competitive Pressure on Autonomic Cardiac Activity among Elite Orienteering Athletes

**DOI:** 10.3390/medicina60091547

**Published:** 2024-09-21

**Authors:** Recep Gorgulu, Hilal Oruç, Cristian Vasile, Ionuț Corlaci, Florin Voinea

**Affiliations:** 1 Psychology of Elite Performance Laboratory (PePLaB), Faculty of Sport Sciences, Bursa Uludag University, Bursa 16059, Turkey; 2 Department of Physical Education and Sports, Faculty of Sport Sciences, Bursa Uludag University, Bursa 16059, Turkey; hilaloruc@uludag.edu.tr; 3 Educational Sciences Department, Faculty of Letters and Sciences, Petroleum-Gas University of Ploiesti, 100680 Ploiesti, Romania; clinical_psycho@yahoo.com; 4 Physical and Sports Education Department, Faculty of Physical Education and Sport, National University of Physical Education and Sports, 060057 Bucharest, Romania; ionut_corlaci@yahoo.com; 5 Department of Physical Education, Sport and Physiotherapy, Faculty of Physical Education of Sport, Ovidius University of Constanta, 900470 Constanta, Romania; voineaflorin09@yahoo.com

**Keywords:** heart rate variability, autonomic nervous system, orienteering, pressure, error, performance

## Abstract

*Background and Objectives*: Orienteering is a sport characterized by high physical exertion and intense mental demands, which increase susceptibility to errors. Understanding the impact of such errors on psychophysiological responses, particularly on heart rate variability (HRV), is essential. This study aimed to investigate the relationship between psychophysiological indicators and checkpoint errors made by elite orienteers during official competition. *Materials and Methods*: Fifty-three orienteers participated in this study, and their performance was continuously monitored and recorded by using a global positioning system (GPS) and HRV data. Errors made during the orienteering events were identified and analyzed. HRV data were examined in three temporal segments: before, during, and after the identified and standardized errors. *Results*: The analyses indicated that errors significantly impacted HRV indices across multiple domains: the time domain, frequency domain, and nonlinear domain. Additionally, a significant effect of sex on the normalized the root mean square of successive differences (r-MSSD) before and after the error was observed. *Conclusions*: The findings of this study underscore the significant impact of errors made by orienteers on cardiovascular responses, as evidenced by measurable alterations in HRV metrics. Cardiovascular activity, represented by the HRV, can provide useful information for coaches and sport psychologists to adopt effective training programs for athletes.

## 1. Introduction

In competitive sports, psychophysiological functions are essential for understanding human performance under extreme conditions, especially in outdoor sports such as orienteering. Orienteering is an endurance event that involves a cross-country time trial between various checkpoints. Similar to other endurance sports, the primary objective in orienteering is to complete the race in the shortest possible time by minimizing or avoiding errors at the checkpoints. Therefore, it stands apart from other running sports due to its unique psychophysiological aspects, particularly in processing visual information and navigating the type of terrain encountered [1,2]. During competitions, orienteers run at high speeds [3] while simultaneously handling challenging cognitive tasks such as decision-making [2,4] and emotion regulation. Accordingly, elite-level orienteers not only have highly aerobic capacities [1,5] but also excellent cognitive ability to navigate while running through unknown terrain. Beyond aerobic capacity, the mental processes of finding the best route between checkpoints while avoiding checkpoint errors are crucial for success in orienteering [6]. For example, someone with high aerobic capacity may experience a delay in the race’s total time due to an error in finding the correct checkpoints under pressure. This happens more frequently if individuals are more attuned to keep in mind previous errors and appraise them as likely to influence subsequent performance [7]. As a result, athletes must withstand compounded psychophysiological demands that can affect both psychological and physiological responses, potentially hindering performance, particularly in competitive settings [8,9]. Earlier studies exploring the relationship between athletes’ simultaneous cognitive and physical performance have primarily focused on laboratory-based research, which emphasizes the impact of dual-task interference. The extended duration of the race, coupled with the sport’s high cognitive demands, positions orienteering as an ideal model for studying the psychophysiological demands of performance [10].

### 1.1. The Role of the Autonomic Nervous System in Orienteering

A key system involved in the generation and regulation of these psychophysiological demands is the autonomic nervous system (ANS). The ANS is generally thought to have two major branches—the sympathetic nervous system, which is associated with energy mobilization, and the parasympathetic nervous system, which is associated with vegetative and restorative functions; these are the most prominent determinants of cardiac function, such as heart rate [11]. Moreover, the ANS is the primary response to the stress experienced by the body during exercise. Once the stressor is removed, the parasympathetic nervous system takes over, lowering blood pressure by reducing heart rate. In this scenario, the sympathetic nervous system is activated, while the parasympathetic nervous system is inhibited [12].

Modulation of the ANS during precision events such as orienteering competitions (e.g., trying to find the best route in an orienteering competition) can be explored by monitoring heart rate variability (HRV), which is a measure of beat-to-beat variation in R-R interval length that provides an indicator of autonomic control of the heart [13,14]. HRV is a simple and noninvasive measurement of interactions between the sympathetic and parasympathetic nervous systems that can be derived from data collected by heart rate monitors [15]. Hence, HRV has attracted an appreciable amount of scientific interest from researchers in the fields of exercise physiology, sports science, and medicine due to the technological development of high-specification heart rate monitors and appropriate analytical software [16,17,18]. Moreover, the HRV is a noninvasive, economic, and simple measurement that has attracted the attention of many researchers [19]. Investigating how HRV changes as a function of human performance provides important insights into the relationships between psychological and physiological processes. For example, an orienteer runs through a target at a high velocity under time pressure when his/her cardiac activity is relatively high, and suddenly realizing an error in relation to the previous checkpoint may cause him/her incremental state anxiety [2] and therefore an acute decrease in HRV [20], which may negatively impact performance by slowing down or causing him/her to become confused.

Clinical evidence has demonstrated that HRV parameters are responsive to variations in parasympathetic activity related to psychological factors [21,22,23,24]. More specifically, research has demonstrated that the ANS, represented by HRV, has the potential to adapt not only to training loads on aerobic capacity but also to competitive stress (e.g., state anxiety, cognitive load, time pressure) [25,26], which can be useful information for coaches and sport psychologists to adopt effective training programs for their athletes. HRV analysis is emerging as an important tool for assessing several mental states, for example, emotion regulation [27], which is vital for athlete well-being and athletic performance [28]. Emotion regulation heavily relies on an individual’s capacity to adjust their physiological arousal in real time [29]. Consequently, analyzing HRV can enhance our understanding of the psychological–and physiological dynamics involved in emotion regulation [30]. For example, an orienteering athlete who reaches a checkpoint and realizes the correct order of the targets on the map may feel calm and relaxed. However, in contrast, an athlete may feel anxious and jittery due to realizing an error in the order of the targets that would cause a costly delay in the race. Researchers have claimed that HRV patterns are sensitive to changes in mental states [31]. Adding a mental load to a physical task further impacted HRV parameters related to autonomic cardiac regulation. Taelman and colleagues [31] explored whether introducing a mental load during physical tasks affected HRV parameters associated with autonomic cardiac modulation, finding that HRV patterns decreased from low to high mental loads. Low HRV is linked to various psychological and physical issues and is considered a biomarker for diminished self-regulatory capacities in physiological, emotional, and cognitive responses, as well as reduced adaptability to environmental stress and demands [27,32,33]. Recent research has employed HRV analysis to examine anxiety and stress across various sports settings, including swimming and golf [25,34,35,36]. Conversely, clinical studies have shown that mental stress increases sympathetic activity while decreasing parasympathetic activity [37,38]. For instance, Dishman and colleagues [37] found lower cardiac vagal components of HRV in normotensive individuals with higher levels of trait anxiety and perceived stress. Hjortskov and colleagues [38] assessed anxiety and stress under combined physical and mental workloads, concluding that HRV is a more effective indicator of mental stress compared to blood pressure.

### 1.2. Limitations of Previous Studies

Considering the above-cited studies, researchers have largely focused on HRV responses before or after predominantly physically demanding tasks, mainly laboratory-based investigations. However, despite the evidence documenting the link between mental workload and HRV [11,39], few previous studies have reported results related to HRV responses before and after a task requiring the use of cognitive skills in addition to demanding physical skills such as archery [40,41], golf putting [42], air-pistol shooting [43], and riffle shooting [44]. To the best of our knowledge, there are no studies examining these combined skills, which are a unique athletic discipline in which perceptual motor skills may have a crucial effect on performance during competition, specifically in orienteering. Thus, there is a clear need to examine orienteers’ HRV responses during competition, especially in relation to the errors they make. Knowing the relationship between cardiovascular activities via HRV and making errors in orienteering would be an essential aspect of every specialist working with orienteering athletes.

Another issue that deserves attention is whether ANS may differ according to sex under such competitive conditions. Although orienteering is not a mixed-gender sport, the competitive conditions and psychophysiological demands of the sport are the same across genders. Therefore, differences in the ANS according to sex have not been well documented; investigating the effects of sex on the ANS may prevent misinterpretation of the results [45]. A comparison of ANS scores by sex could improve the accuracy of the data obtained in the present study. Therefore, the present research sought to examine the effect of gender on orienteers’ ANS responses when they make an error. Orienteering is an excellent model for examining the interaction between performance and cardiovascular responses such as heart rate variability. Orienteers require advanced map-reading skills, racing ability, and physical fitness. However, there is a need for studies examining the physiological and cognitive demands placed on these athletes, as well as research into how these attributes contribute to performance improvement [46].

### 1.3. The Current Study

Enhancing objective information about an orienteer’s route between checkpoints, especially when there is an error, in combination with psychophysiological HRV data could be an objective and robust method for studying performance enhancement in such endurance sports. To the best of our knowledge, the effects of autonomic cardiac activity on orienteering athletes during competition have never been evaluated. Specifically, no study has examined (1) cardiac activity via HRV just before, during, or after an error at the checkpoint of the race and (2) the influence of sex differences on autonomic cardiovascular activity under pressure. Thus, the main aim of the current study was to investigate the cardiac activity of elite orienteers during competition, while the latter aim was to understand the relationship between sex differences and cardiac activation, especially when there is an error under pressure. Therefore, we hypothesize that there will be certain differences in orienteers’ cardiac activity during different stages of the competition and that there will be some cardiac changes based on sex, especially when there is an error.

## 2. Materials and Methods

### 2.1. Participants

To meet the inclusion criteria for this study, we required participants to be older than 18 years and to have completed the full distance of the official orienteering event organized by the Turkish Orienteering Federation. A total of 53 elite orienteering athletes (32 men, 21 women; Mean_age in years = 24.85, SD = 5.70; Mean_height_centimeters = 171.97, SD = 8.10; Mean_weight_kilograms = 62.72, SD = 10.11) provided data during the race. We recruited participants on a volunteer basis through sports clubs officially participating in orienteering races organized by the Turkish Orienteering Federation. The participants had at least 5 years of running experience at official orienteering events at the national or international level. This ensured that all participants were familiar with the procedures and techniques of orienteering and, consequently, could provide valid data for the current study. To qualify for inclusion in this study, the athletes had to fulfill the following criteria: (a) be a non-smoker orienteering athlete for at least five years; (b) systematically train at least 3 times per week; (c) be competed in the middle- or long-distance event of the orienteering championships, organized by the Turkish Orienteering Federation; and d) participate in HRV measurements during the race.

The sample size of this study was determined by using G-Power 3.1. Faul and colleagues [47] calculated the sample diameter as *n* = 50 with 95% confidence, 0.5 sensitivity, and 80% power (1 − *β* = 0.80). Written informed consent was obtained from the participants, and this study was carried out in accordance with the Declaration of Helsinki. The protocol was approved by the local institution for the current study (Bursa Uludağ University, Faculty of Medicine Clinical Research Ethics Committee, Ethics number: 2020-19-2, date: 13 November 2020).

### 2.2. Measures

#### 2.2.1. Orienteering Competition and Categorization

The orienteering races included in this study were officially recognized by the Turkish Orienteering Federation. Each race featured an average winning time exceeding 35 min, thus categorizing them as ‘middle distance’ events, excluding sprint orienteering competitions. Each participant competed in one of the races held throughout the year, which took place in various terrains characterized by technical features. This study focused on the W/M 20 and 21 categories, as these included experienced participants who had been involved in orienteering for at least five years.

#### 2.2.2. Data Collection and Extraction

The data were derived from the competition records of athletes who participated in a range of official events listed in the 2021–2022 activity calendar of the Turkish Orienteering Federation. This included terrain-based events where men (*n* = 32) and women (*n* = 21) competed. The authors systematically examined each athlete’s race, identifying and recording errors made at each control point. These errors included visible deviations from the route, searching around the control point, and missing the control point. It is important to note that the data collection focused solely on the most obvious errors confirmed by the athlete. This approach allowed for standardizing an athlete’s errors, excluding less obvious ones. A standardized obvious error for a performance by a total of 53 elite orienteering athletes was reported within the specified time frame.

#### 2.2.3. Heart Rate Variability Data Were Collected

HRV data were obtained by wearing a Polar V800 monitor (Polar Electro Oy, Kempele, Finland) and an H10 chest strap. The data were saved as RR interval data files, with intervals of milliseconds the data obtained from the watches were subsequently transferred to a computer, and the Polar HRM raw unfiltered RR data was exported from the Polar Flow web service as a space delimited.txt file. The raw, unfiltered RR data from the Polar HRM were exported from the Polar Flow web service as a space delimited .txt file. Ectopic beats and artifacts (<3%) were automatically detected and corrected according to the manufacturer’s guidelines to create a normal-to-normal (NN) interval time series [48]. HRV parameters were computed using Kubios HRV Premium version 3.5.0 [48] for offline analysis. HRV analysis included time domain, frequency domain, and nonlinear-domain measurements. Time domain indices comprised the standard deviation of all normal R-R intervals (SDNN) and the root mean square of successive normal R-R interval differences (r-MSSD). Frequency domain calculations were performed using the Fast Fourier Transform based on Welch’s periodogram method (window width: 300 s; window overlap: 50%; frequency domain points: 300 points/Hz). Frequency domain indices are presented as absolute (ms^2^), normalized to total power (nu), and logarithmically transformed absolute (log ms^2^) values.

The normalized total power LF-to-HF (LF/HFnu) ratio was used to quantify the sympathovagal balance. Additionally, the SD1/SD2 ratio was calculated as part of the nonlinear domain of HRV. Time domain measurements are closely correlated with parasympathetic nervous system (PNS) activity and are used to evaluate vagal activity. LF/HF is accepted in frequency domain measures to reflect the sympathovagal balance [49]. SD1/SD2 is correlated with the LF/HF ratio, while SS and SS/PS are new indices introduced to enhance the physiological interpretation of HRV based on the Poincaré plot analysis method [50].

#### 2.2.4. Collection of GPS Data

GPS data were obtained by wearing a Polar V800 monitor (Polar Electro Oy, Kempele, Finland). The data retrieved from the monitor were exported as GPX or ZIP package files via Polar Pro Trainer 5 software (Polar^®^ ProTrainer, Kempele, Finland) for the routes. The received GPS data were subsequently transferred to the Livelox web-based application for analysis. The Livelox web service is an analytics tool for orienteering that provides visualization of GPS tracks and route options on a map.

#### 2.2.5. Standardization of HRV Data

The errors made by each athlete were conceptualized by monitoring their GPS data and limiting them to one-minute examples, considering obvious errors. The participants’ race data were matched with GPS data from the Polar V800 and analyzed by the authors. The authors examined the tracks of the participants via the Livelox web service and identified the errors. When the participants were interviewed, the errors identified by the authors were consistent with those reported by the participants. Errors specifically consisted of visibly off course, going to the wrong destination checkpoint, passing the checkpoint, or being searched near the checkpoint circle. One-minute samples were analyzed for errors made by the athletes one minute before the error and one minute after the error via o Kubios HRV Premium software version 3.5.0 (Biosignal Analysis and Medical Imaging Group, Department of Physics, University of Kuopio, Kuopio, Finland, [48]).

### 2.3. Procedure

**Figure 1:**
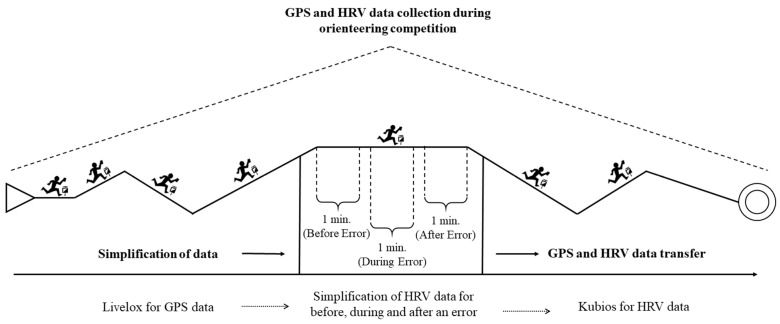
Demonstration of procedure of the study.

The researchers contacted orienteering clubs and the National Orienteering Federation. The procedures and objectives of this study were thoroughly explained, and permission was sought to collect data during the competition season. HRV and GPS data were collected at the middle-distance competition site (Figure 1). In the first stage, the participants wore a Polar V800 monitor and a Polar H10 chest strap 10 min before the competition. Each participant started the monitor for recording before the competition, and it continued throughout the competition. In the end, we removed the monitor and chest band from the participants.

### 2.4. Statistical Analysis

Statistical analysis was performed using the Statistical Package for the Social Sciences (IBM SPSS Statistics Inc., Chicago, IL, USA). After checking the normality of the distribution with the Shapiro–Wilk test, descriptive statistics were used to examine the means and standard deviations of the demographic and participant characteristics (Table 1). We first conducted a series of repeated measures analyses of variance (ANOVAs) to explore whether the HRV indices of the SDNN, r-MSSD, LF/HF, and SD1/SD2 groups differed significantly before, during and after making standardized errors for the entire sample.

Therefore, the equality of variances between data points was evaluated using Mauchly’s test of sphericity. Results are presented as mean ± SD. If sphericity was violated, the Greenhouse–Geisser correction was applied. In cases where significant differences were found in the within-subject effects test, post-hoc comparisons using Bonferroni’s method were conducted to identify pairwise differences. Statistical significance was set at *p* < 0.05.

## 3. Results

The descriptive statistics are shown in Table 1. The sex differences in the participants are presented as the mean, standard deviation, and minimum and maximum values for age, height (cm), weight (kg), and BMI (kg/m^2^).

After scrutiny by one of the authors, who is a national team orienteering athlete, the data for 53 orienteers were deemed suitable for detailed analysis. The descriptive statistics of the HRV indices for the time domain (SDNN, r-MSSD, and MeanHR), frequency domain (LF/HF), and nonlinear (SD1/SD2) domain parameters are shown in Table 2. When considering the entire sample, repeated measures analysis of variance (ANOVA) indicated that SDNN [F(2,102) = 8.252, *p* = 0.000, ηp^2^ = 0.139, observed power = 0.854], r-MSSD [F(2,102) = 4.077, *p* = 0.020, ηp^2^ = 0.074, observed power = 0.713], and MeanHR [F(2,102) = 4.272, *p* = 0.017, ηp^2^ = 0.077, observed power = 0.734] for the time domain differed significantly before, during, and after periods of standardized error at the competition. A marginally significant difference was observed in the LF/HF band [F(2,102) = 2.995, *p* = 0.054, ηp^2^ = 0.055, observed power = 0.570] in the frequency domain, and a significant difference was observed in the SD1/SD2 band [F(2,102) = 4.291, *p* = 0.016, ηp^2^ = 0.078, observed power = 0.736] of the nonlinear measure before, during, and after periods of standardized error during the competition.

Despite the significant within-subject effects in terms of SDNN, the r-MSSD, mean HR, LF/HF, and SD1/SD2 paired sample *t*-tests with Bonferroni correction failed to find any significant differences before or during the competition. However, the SDNN decreased from before to after the error was made [*t*(40) = 2.47, *p* = 0.004]. No significant differences were found between during and after the error was made for the r-MSSD [*t*(45) = −1.59, *p* = 0.059]. On the other hand, the mean HR increased from before to after the error was made [*t*(52) = −2.98, *p* = 0.002]. In addition, there was no significant difference in LF/HF before or during the error [*t*(52) = −0.508, *p* = 0.307]. However, there was a significant difference in LF/HF during and after making the error [*t*(52) =2.18, *p* = 0.017] as well as before and after making the error [*t*(52) = 1.94, *p* = 0.028]. Similarly, there was no significant difference in the SD1/SD2 before or during the error [*t*(37) = −223, *p* = 0.412], but there was a significant difference in the SD1/SD2 during and after the error [*t*(37) = 2.42, *p* = 0.010] or before and after the error [*t*(38) = 2.47, *p* = 0.009].

One-way MANOVA was conducted to explore whether male and female athletes had different HRV response patterns before, after, and after the error. HRV variables normalized to baseline were entered into the model as the dependent variables. The independent variable was sex. The MANOVA results demonstrated no difference in terms of normalized SDNN values between male and female orienteering athletes. According to Pillai’s trace, the MANOVA revealed no significant effect of sex before or during the r-MSSD. However, there was a significant difference before and after the error for only male athletes. According to Pillai’s Trace (*p* < 0.01), the MANOVA revealed a significant effect of sex on the normalized r-MSSD before and after the error was made [V = 0.153, F(3,31) = 1.86, *p* = 0.01, ηp^2^ = 0.15, observed power = 0.43]. Therefore, compared with those of their female counterparts, the HRV parameters of male orienteering athletes were significantly lower before and after making the error.

## 4. Discussion

In the present research, we sought to examine the effects of situational pressure and making errors on subsequent performance considering potential changes in the ANS in real-life settings with high ecological validity using play-by-play data generated via the Livelox software. In particular, we aimed to test the autonomic cardiac activity of elite orienteering athletes before, during, and after making errors during competition. The latter objective of this study was to evaluate the effect of sex on orienteering athletes’ autonomic cardiac responses in relation to errors made during competition. The rationale for selecting orienteering as an endurance sport is that it demands both physical attributes and mental skills, potentially leading to distinct HRV responses compared to other physical activities like resistance training and cycling.

In line with our first hypothesis, there was a clear interaction effect of pressure and an error was made that would have multiplicative effects on performance, with objective and robust evidence emanating from HRV in three different time domains namely time, frequency, and nonlinear domains. First, we hypothesized that there would be a significant difference in the time domain HRV parameters (SDNN and r-MSSD) of the athletes before, during, and after making an error during the orienteering race. Our results provide evidence that the r-MSSD (root mean squared difference) is significantly lower after an error is made during an orienteering competition. The r-MSSD is a time-domain measure of heart period (HP) variability that is particularly sensitive to short-term, high-frequency fluctuations in HP [51,52]. In addition to r-MSSD, one of the simplest time-domain analysis variables is the standard deviation of the NN interval (SDNN), which measures the variability of NN intervals. A higher and more irregular HRV results in an increased SDNN. Thus, SDNN serves as an indicator of physiological resilience to stress [53]. As for the r-MSSD, our results provide support for the notion that the SDNN significantly decreases after elite orienteering athletes are mistakenly handled under pressure. In addition, our results revealed an increase in the LF/HF ratio, a frequency domain measure of HRV, in male athletes, especially after an error during the competition. Psychological stress was significantly linked to an increase in the LF/HF ratio, indicating heightened sympathetic nervous system activity during stressful times of the day [54]. This can be explained by the fact that male-orienteering athletes are more prone to making an error during a competition than their female counterparts. This result is also supported by the idea that male athletes can be more competitive and glory-seeking than their female counterparts. Finally, for the nonlinear domain of HRV, SD1/SD2 significantly differed after making an error for both male and female athletes. Taken together, the results of the present study provide support for the temporal and partial support for the frequency and nonlinear domains of HRV and indicate that the HRV indices of elite orienteering athletes rapidly decrease, especially after an error. The observed effect of pressure on performance is in line with prior work related to a decrease in cardiac activity, which was observed immediately after a performance-related error [55,56]. HRV is frequently associated with the stress response because of its connections to the ANS, especially the PNS [11,19].

Moreover, increasing LF/HF values during the error and decreasing SDNN and r-MSSD values before and after the error are consistent with previous findings utilizing HRV as an indicator of a successful physiological response to anxiety [57,58]. Examining elite orienteering athletes’ cardiac activity as an indicator of the ANS during official competition reveals the emotional state of the athletes, especially when they make a sudden error. Like in our study, scholars found that the HRV values (time domain measures) of the participants in the high-anxiety condition were lower than those in the low-anxiety condition, and low values indicate a high-stress response [35,43,59].

Throughout the day, the heart frequently slows down and speeds up in response to various activities—whether waiting at traffic lights, making an error while playing the piano, exercising, meditating, or experiencing a sudden scare. The pattern of cardiac activity is known to vary with environmental demands and is modulated by sympathetic and parasympathetic activity, which adaptively accelerates or decelerates heart rate based on the situation [60,61,62,63]. This phenomenon has been explored in the psychophysiological literature in various contexts, including reactions to novel stimuli, recovery after task errors, and defensive responses to threats. Cardiac activity is finely tuned by sympathetic and parasympathetic influences, shaping both perception and action tendencies. Most research on the relationship between cognitive state and HRV has relied on a single HRV index as the dependent variable [28,39,64] or manipulated one HRV index as an independent variable [39]. Our results indicate that while time and frequency domain measurements are strongly correlated, they may not precisely reflect the same physiological substrate. It is well established that HRV can increase due to elevated parasympathetic or sympathetic tone or both [65]. Our findings suggest that an increase in perceived cognitive load (e.g., making a checkpoint error in orienteering) elevates both sympathetic and parasympathetic components of the ANS. Mental stress has been shown to raise blood pressure [66], and an increase in blood pressure in young, healthy individuals could be expected to enhance parasympathetic tone through the baroreceptor reflex mechanism.

The results of the present study indicated that endurance-based performance requiring perceptual motor skills under pressure might lead to different HRV responses in elite orienteering athletes. However, the initial aim of the present study was to understand the differences in HRV patterns based on sex in relation to making an error during an orienteering competition under pressure. As shown in the present study, female orienteers had a significantly greater LF/HF ratio than male orienteers did immediately during the error. This finding indicates a lower cardiac vagal component of HRV among men and women who perceive more stress due to performance failure. On the other hand, we found that the r-MSSD values observed before and after the error significantly changed before and after the error for the male orienteering athletes only (*p* < 0.01). Additionally, previous research revealed that decreased r-MSSD has previously been associated with elevated precompetitive anxiety [35,36,57,59]; therefore, monitoring HRV components may help to control the psychological state after making such errors during pressurized competitive sporting events (e.g., orienteering). Therefore, coaches and sport psychologists, aiming to improve orienteering performance, may consider monitoring HRV during their training sessions. Future research should consider sex differences when performing HRV studies. These results suggest that under competitive settings involving situational pressure and task difficulty, male and female orienteering athletes may not have similar autonomic cardiac responses during orienteering competition. However, previous studies have shown contradictory results under different conditions. For example, Tok and colleagues [41] did not find any difference between male and female archers during the shooting and recovery periods. However, in their study, they did not specifically focus on the standardized period of HRV for error-based performance. Accordingly, in the current study, we were unable to control the menstrual cycles of female athletes during the data collection procedure, which may have had a vital effect on the changes in HRV, as alterations in the balance of ovarian hormones in female athletes can influence autonomic cardiac activity [67].

Overall, this study demonstrated that short-term psychological stress could lead to a significant increase in heart rate and a general decrease in ANS activity, as indicated by a reduction in the standard deviation of normal interbeat intervals. Additionally, within the time domain measures of HRV, the r-MSSD (*p* < 0.01) and SDNN (*p* < 0.000) were significantly lower. These short-term changes in HRV reflect rapid alterations in the PNS.

Different techniques are available for measuring HRV; these include time domain, frequency domain, and nonlinear measures [13,14,68]. Although frequency domain techniques have the advantage of providing more direct quantification of the ANS [69], importantly, time domain measurements are strongly influenced by changes in both sympathetic and parasympathetic activity, making them nonspecific measures of autonomic modulation [30]. As one of the time domain measures of HRV, the r-MSSD is the most sensitive parameter, especially for short-duration recordings [70]; therefore, the r-MSSD could be a robust and valid indicator of emotional state in competitive situations, similar to our research. For the frequency domain measures of HRV, the ANS tends to increase sympathetic activity while inhibiting parasympathetic activity in a competitive stress situation. For example, under pressurized competition, the LF parameter and the LF/HF ratio increase, indicating a predominance of sympathetic activity according to previous studies [24,25,71]. However, researchers generally reject claims that HRV reflects sympathetic activity or the sympathovagal balance accordingly [72,73,74,75,76,77]. This is because the ‘sympathovagal balance’ is arguably calculated with the LF/HF ratio.

Mathematical manipulations of these frequency domain measures, such as the SD1/SD2, are also used and correlated with the LF/HF ratio [78,79], in which higher LF/HF ratios putatively reflect relatively greater sympathetic nervous system (SNS) activity. However, contradictory to the initial claim, it has been proposed that low-frequency HRV is not a reliable indicator of SNS [72,80,81], although debate exists about this distinction. The mechanism underlying whether HRV parameters reflect the balance between sympathetic activity and parasympathetic activity in competitive situations is still not well understood. Therefore, the current study offers preliminary evidence suggesting that physical activities requiring mental and attentional skills, such as orienteering, can produce HRV responses distinct from those observed in other physical activities. The different HRV responses seen in orienteering athletes, such as increased parasympathetic activity during checkpoint errors, may be related to the connection between cardiac vagal tone and cognitive performance [82,83,84]. Therefore, it could be concluded that elite orienteering athletes’ HRV responses may differ from those of other athletes mainly due to the cognitive and perceptual demands of the sport, such as middle or long-distance running, contact with the map and terrain, and checking the correct direction while running. Therefore, further studies in relation to human performance requiring mental and attentional qualities (e.g., finding the best route in orienteering) can give rise to HRV responses that are different in other sports, such as running, cycling, and resistance training, relatively with less cognitive demands due to the nature of the sport.

This study is one of the first analyses of performance error-based psychophysiological investigations of elite orienteering athletes; however, it is not without its limitations. First, we only collected data from middle-distance competitions; however, in orienteering, there is also a short-distance type of event called a “sprint’’. Therefore, in relation to cardiac activity, it would be interesting to evaluate elite orienteering athletes’ performance in short-distance events under time-related pressure. Second, we did not control for participants’ respiration rates due to technical and environmental restrictions. Therefore, as respiration may affect HRV, in future studies, the effect of respiration should be taken into consideration in a similar design of orienteering competition settings. Third, we failed to control for the quality of sleep, as there may be a strong correlation between quality of sleep and changes in HRV. Therefore, future research should take this into account and may use self-reported sleep quality questionnaires or inventories to address this issue.

## 5. Conclusions

The results of the current study may also have several implications for orienteering coaches, athletes, and sport psychologists. In orienteering, the focus is always on the general result of the performance. A small error—for example, missing the first control point or losing contact with the map and the terrain—may result in an important loss of time. Knowing the relationship between cardiovascular activities via HRV and making errors can be key to success in orienteering, an essential aspect for every specialist working with orienteering athletes. The data analyses revealed that errors significantly impacted the HRV indices across multiple domains: the time domain (meanHR, SDNN, and r-MSSD), frequency domain (LF/HF), and nonlinear domain (SD1/SD2). Regarding sex-related differences, a significant effect was found (only for male athletes) on the normalized r-MSSD before and after the error. Incorporating HRV monitoring and improvement strategies into training regimens could enhance athletic performance by offering deeper insights into the psychological stress or competitive anxiety associated with performance decrements. These insights may facilitate the development of cognitive training programs, such as deep breathing and HRV biofeedback training, ultimately contributing to improved orienteering performance.

## Figures and Tables

**Table 1:** Descriptive statistics of participants’ demographic information.

Gender	*n*	Variable	x̄	SD	Min	Max
Female	21	Age (years)	23.17	3.92	19.00	30.00
		Height (cm)	164.64	5.61	155.00	178.00
		Weight (kg)	52.88	4.01	46.00	60.00
		BMI (kg/m^2^)	19.48	1.04	17.50	21.30
Male	32	Age (years)	26.08	6.54	18.00	40.00
		Height (cm)	177.39	4.62	170.58	186.00
		Weight (kg)	70.00	6.28	58.00	80.00
		BMI (kg/m^2^)	22.22	1.63	18.70	25.10
Total	53	Age (years)	24.85	5.70	18.00	35.00
		Height (cm)	171.97	8.10	155.00	186.00
		Weight (kg)	62.72	10.11	46.00	80.00
		BMI (kg/m^2^)	21.06	1.96	17.50	25.10

Note: cm = centimeters; kg = kilogram; BMI (kg/m^2^) = kilogram/meter square.

**Table 2:** Comparison of HRV variables across three different time points by sex.

Variables	Gender	Before (x̄)	*SD*	During (x̄)	*SD*	After (x̄)	*SD*	F	*p*	η_p_^2^	Power
MeanHR (bpm)	Female	164.58	17.41	167.90	16.06	168.91	15.02	4.272	0.017 *	0.077	0.734
	Male	165.26	18.27	164.98	18.05	169.84	12.66				
	Total	164.99	17.77	166.14	17.19	169.47	13.51				
SDNN (ms)	Female	6.55	6.61	3.86	1.63	3.66	1.59	8.252	0.000 ***	0.139	0.854
	Male	6.27	5.21	4.36	2.40	4.09	2.62				
	Total	6.38	5.74	4.16	2.13	3.92	2.26				
r-MSSD (ms)	Female	5.70	7.08	3.63	1.85	4.03	1.97	4.077	0.54	0.055	0.570
	Male	7.09	6.84	4.89	2.92	5.32	3.68				
	Total	6.54	6.90	4.39	2.61	4.81	3.16				
LF/HF (%)	Female	4.56	4.86	6.15	9.10	3.39	4.15	2.995	0.020 *	0.074	0.713
	Male	3.29	5.58	3.00	3.65	1.59	1.61				
	Total	3.79	5.30	4.24	6.50	2.30	2.99				
SD1/SD2 (%)	Female	1.94	1.27	2.00	1.32	1.55	0.70	4.291	0.016 *	0.078	0.736
	Male	1.63	1.01	1.58	1.02	1.20	0.36				
	Total	1.75	1.12	1.74	1.15	1.34	0.55				

Notes: * *p* < 0.05, *** *p* < 0.001. Smoothness Priors Analysis (SPA) technique applied to the time series of HRV data with Kubios HRV Premium software version 3.5.0.

## Data Availability

The raw data supporting the conclusions of this article will be made available by the authors without undue reservation.
